# KPNA2 promotes angiogenesis by regulating STAT3 phosphorylation

**DOI:** 10.1186/s12967-022-03841-6

**Published:** 2022-12-28

**Authors:** Yujie Jia, Qi Wang, Minglu Liang, Kai Huang

**Affiliations:** 1 Clinic Center of Human Gene Research, Union Hospital, Tongji Medical College, Huazhong University of Science and Technology, 1277 Jiefang Ave., Wuhan, 430022 China; 2 Hubei Key Laboratory of Metabolic Abnormalities and Vascular Aging, Huazhong University of Science and Technology, Wuhan, 430022 China; 3 Hubei Clinical Research Center of Metabolic and Cardiovascular Disease, Huazhong University of Science and Technology, Wuhan, 430022 China; 4 Department of Cardiology, Union Hospital, Tongji Medical College, Huazhong University of Science and Technology, Wuhan, 430022 China; 5 Department of Urology, Union Hospital, Tongji Medical College, Huazhong University of Science and Technology, Wuhan, 430022 China

**Keywords:** KPNA2, STAT3, Endothelial, Angiogenesis, Hindlimb ischemia

## Abstract

**Purpose:**

Angiogenesis is involved in many pathological and physiological processes and is mainly driven by hypoxia. Karyopherin subunit alpha 2 (KPNA2), a member of the nuclear transport protein family, was recently shown to be induced by hypoxia in various types of tumours, so we aimed to investigate the role and mechanism of KPNA2 in angiogenesis under hypoxia.

**Materials and methods:**

After overexpression or knockdown of KPNA2 in human umbilical vein endothelial cells (HUVEC) by adenovirus vector infection, the tube formation, proliferation and migration of HUVEC under hypoxia were detected by tubule formation assay, 5-ethynyl-2′-deoxyuridine (EdU) staining and Transwell assay, respectively. After overexpression or knockdown of KPNA2 in a murine hindlimb ischemia model by local injection of purified adenovirus vector into the gastrocnemius muscle, blood flow changes were examined with a laser Doppler system. Changes in KPNA2-binding proteins under hypoxia were detected by immunoprecipitation-mass spectrometry (IP-MS) and co-immunoprecipitation (Co-IP). The effect of KPNA2 on signal transducer and activator of transcription 3 (STAT3) was detected by Western blotting and quantitative RT‒PCR.

**Results:**

KPNA2 was upregulated in the HUVEC hypoxia model and murine hindlimb ischemia model. Overexpression of KPNA2 increased the proliferation, migration and tube formation of HUVEC under hypoxia, while knockdown of KPNA2 reduced the proliferation, migration and tube formation of HUVEC. Overexpression of KPNA2 promoted the restoration of blood flow in the murine hindlimb ischemia model, while knockout of KPNA2 inhibited the restoration of blood flow in the murine hindlimb ischemia model. Mechanistically, hypoxia promoted the binding of STAT3 to KPNA2. Overexpression of KPNA2 promoted STAT3 phosphorylation and then upregulated vascular endothelial growth factor (VEGF) and angiopoietin 2(ANGPT2), whereas knockdown of KPNA2 inhibited STAT3 phosphorylation and then downregulated VEGF and ANGPT2.

**Conclusion:**

Our study demonstrates that hypoxia promotes the binding of STAT3 to KPNA2 and KPNA2 promotes angiogenesis under hypoxia by promoting the binding of STAT3 and JAK1 and regulating STAT3 phosphorylation.

**Supplementary Information:**

The online version contains supplementary material available at 10.1186/s12967-022-03841-6.

## Introduction

Angiogenesis refers to the process of generating new blood vessels and is strictly regulated by angiogenic factors and cytokines. Angiogenesis plays a central role in tissue repair, wound healing, and organ development [1]. Abnormal angiogenesis status is associated with many diseases [2, 3]. Excessive angiogenesis aggravates the progression of tumours, diabetic retinopathy, arthritis, and other diseases, while insufficient angiogenesis is detrimental to recovery from diseases such as peripheral arterial disease (PAD), myocardial infarction, and stroke [4, 5].

Hypoxia is characteristic of many ischemic diseases and tumours and is a strong stimulus for angiogenesis [6]. Hypoxia drives angiogenesis primarily by inducing the release of proangiogenic growth factors [4, 7]. Vascular endothelial growth factor (VEGF) is considered a major angiogenic growth factor and the main target of antiangiogenic therapy [8]. However, VEGF pathway inhibitors are failing to produce enduring clinical responses in most patients and many patients with metastatic disease are refractory or resistant to VEGF inhibitors [5, 9–12]. For ischemic disease, there are few effective treatments other than endovascular therapy [13–15]. Therefore, it is of great importance to identify the key regulators of angiogenesis and therapeutic target pairs in this process.

Karyopherin subunit alpha (KPNA) is a nuclear transport protein family that can bind cargo proteins containing a nuclear localization signal (NLS) and transport them into the nucleus by interacting with karyopherin subunit beta 1 (KPNB1). However, an increasing number of studies have indicated that KPNA has multiple functions beyond nuclear transport [16]. Previous studies suggest that the KPNA family play a role in the angiogenesis of ageing myocardial microvascular endothelial cells and ageing gastric mucosal endothelial cells [17, 18]. Karyopherin subunit alpha 2 (KPNA2), a member of the KPNA family, has been implicated in tumorigenesis and progression in previous studies [19]. KPNA2 can be induced by hypoxia in various tumour cell types [20, 21], and its knockdown inhibits osteosarcoma cell proliferation and reduces blood flow [22]. Therefore, KPNA2 may be a key molecule in hypoxia-induced endothelial angiogenesis, and this hypothesis deserves further study.

In this study, we found that the binding of KPNA2 to signal transducer and activator of transcription 3 (STAT3) was increased under hypoxia, and KPNA2 promoted angiogenesis in vivo and in vitro by regulating the phosphorylation of STAT3 under hypoxia.

## Materials and methods

### Animals

Adult male C57BL/6 J mice (8 weeks), obtained from the Laboratory Animal Center at the Huazhong University of Science and Technology were used in this study. All animal protocols were approved by the Institutional Animal Care and Use Committee of Ethics of Tongji Medical College, Huazhong University of Science and Technology.

### Murine hindlimb ischemia model

As described previously [23], male c57Bl mice (8 weeks) were anesthetized by intraperitoneal injection of pentobarbital sodium (50 mg/kg body weight). After skin preparation, the superficial skin was incised to expose the femoral artery. The femoral artery was separated from the accompanying vein and nerve. The proximal and distal ends of the femoral artery were ligated, and the middle segment was cut to induce hindlimb ischemia.

### Detection of perfusion recovery

Blood perfusion before and after surgery was monitored with a laser Doppler system (Perimed, Sweden). In detail, the mice were anaesthetized by intraperitoneal injection of pentobarbital sodium, and then the hindquarters were imaged by laser Doppler imaging. The reduction in and recovery of blood perfusion were obtained by calculating the ratio of perfusion in ischemic to nonischemic limbs.

### Cell culture

Human umbilical vein endothelial cells (HUVEC) were isolated from fresh umbilical cords as described previously [24]. The cells were cultured in Endothelial Cell Medium (ECM) (ScienCell) at 37 °C in a humidified incubator (Thermo Scientific, 3111) with 5% CO_2_. Hypoxia exposure was applied by culturing the cells in an incubator (Thermo Scientific, 3131) with 1% O_2_ and 5% CO_2_. For endothelial infection, empty adenoviral vector (Ad-Vector) and adenoviruses vector encoding KPNA2 (Ad-KPNA2), scramble short hairpin RNA (Ad-NC), and KPNA2 shRNA (Ad-shKPNA2) were applied to infect HUVEC *in vitr*o. Viral fluid volume = number of cells × multiplicity of infection (MOI) /viral titer. Cells were lysed 48 h after infection.

293 T cells with STR identification were obtained from the American Type Culture Collection. They were cultured in Dulbecco’s modified Eagle medium (DMEM) (Gibco) with 10% FBS (Gibco) and 1% volume of 100 × penicillin/streptomycin solution (Beyotime Biotechnology, C0222). The final concentration of penicillin is 100 U/ml and the final concentration of streptomycin is 0.1 mg/ml. The cells were cultured at 37 °C in a humidified incubator with 5% CO_2_.

### Western blotting

Western blotting was performed as described previously [25]. Tissue or cells were lysed using RIPA lysis buffer (Beyotime Biotechnology, P0013C) supplemented with PMSF (Thermo Scientific, 36978), protease inhibitors (Roche, 04693132001) and phosphatase inhibitors (Roche, 04906837001). Protein concentrations were determined using a Pierce BCA protein assay kit (Thermo Scientific, cat No. 23225). Western blotting was performed with antibodies against the following: KPNA2 (1:1000, Abclonal, A5012), KPNA1 (1:1000, Abclonal, A1742), KPNA3 (1:1000, Abclonal, A8347), KPNA4 (1:1000, Abclonal, A2026), KPNB1 (1:1000, Abclonal, A8610), β-ACTIN (1:1000, CST, 3700S), STAT3 (1:1000, Abclonal, A19566), P-STAT3 (1:1000, Abclonal, AP0705), VEGF (1:1000, Proteintech, 66828–1-Ig), P-VEGFR2 (1:1000, Cell Signaling Technology, #3770), GAPDH (1:1000, Abclonal, AC001), Laminb1 (1:1000, Cell Signaling Technology, #13435), ANGPT2 (1:1000, Abclonal, A11306), Flag (1:1000, Sigma, F2555), JAK1 (1:1000, Abclonal, A18323), JAK2 (1:1000, Abclonal, A7694), SRC (1:1000, Abclonal, A0324), TYK2 (1:1000, Abclonal, A2128), P-JAK1 (1:1000, Abclonal), GPX1 (1:1000, Abclonal, A11166), and MOV10 (1:1000, Abclonal, A7227).

Relative quantitative analysis of protein expression levels was performed using Image Lab software (Bio-Rad).

### Immunofluorescence analysis

Tissue/cells on coverslips were fixed with 4% paraformaldehyde and deparaffinized. Antigen retrieval was carried out by heating. Then, the tissue sections/cells were incubated with donkey serum to block background signal, followed by the detection of specific antigens with primary and fluorescent secondary antibodies. The nuclei were stained with DAPI. Then the tissue sections/cells were observed and photographed under a fluorescence microscope (Olympus). Antibodies against the following were used: CD31 (1:200, Abcam, ab76533), KPNA2 (1:200, Abclonal, A5012), and P-STAT3 (1:200, Abclonal, AP0705). The fluorescent secondary antibodies used are as follows: Alexa Fluor488 donkey anti-rabbit lgG (H + L) (1:400, Life Technologies, A21206), Alexa Fluor555 donkey anti-rabbit lgG (H + L) (1:400, Life Technologies, A31572).

### Recombinant adenovirus production

Recombinant adenovirus was constructed using Gateway cloning according to the manufacturer’s protocol. The entry vector for overexpression is the pENTY-ccDB-T2-pcdh vector with the CMV promoter, and the entry vector for knockdown is the PEnty-kd-ccDb2 vector with the U6 promoter. The Entry clone with the gene of interest flanked by attL sequences was produced using the restriction enzyme and ligase cloning method. Expression clones were generated with Gateway LR Clonas II enzyme mix (Thermo) catalysed in vitro by recombination of the entry vector (containing the gene of interest flanked by the attL sequences) with the destination vector (containing the attR sequences) pDEST (Thermo Scientific). Recombinant adenoviral plasmids were linearized with PacI (Thermo Scientific) and transfected into HEK293A cells for adenoviral packaging and amplification. Then, the adenovirus was purified by CsCl gradient centrifugation.

### Tubule formation assay

Tubule formation assays were performed as described previously [26]. A 96-well plate was coated with 50 µl of Matrigel per well, and then HUVEC were seeded in the Matrigel-coated 96-well plate at a density of 1.5 × 10^4^ cells per well. After 6 h, pictures were taken with a light microscope (Olympus, Tokyo, Japan), and the Angiogenesis Analyser plugin of ImageJ was used to count the total length.

### Transwell assays

Migration assays were performed with a Transwell cell culture insert (3 µm, Corning, NJ). HUVEC (3 × 10^4^ cells) were placed on the upper layer of a Transwell cell culture insert, and ECM was placed below the cell-permeable membrane. Following an incubation period (6 h), the cells that had migrated through the membrane were stained with 0.1% crystal violet for 20 min and counted [27].

### 5-Ethynyl-2′-deoxyuridine (EdU) staining

EdU experiments were performed with a BeyoClick^™^ EdU Cell Proliferation Kit with Alexa Fluor 555 (Beyotime Biotechnology, C0075S). Briefly, HUVEC were seeded in a 96-well plate at a density of 5000 cells per well. The cultured cells were labelled with EdU, fixed, washed, and permeabilized. Click reaction solution (Click Reaction Buffer, CuSO4, Azide 555, Click Additive Solution) was used for the EdU reaction and detection. DAPI was used to stain the nuclei. Fluorescence was observed under a fluorescence microscope (Olympus).

### Extraction of cytoplasmic and nuclear proteins

HUVEC were prepared in 10 cm dishes for extraction of the cytoplasmic and nuclear proteins. Nuclear and cytoplasmic proteins were isolated using NE-PER^™^ Nuclear and Cytoplasmic Extraction Reagents according to the manufacturer’s protocol (Thermo Scientific).

### RNA extraction and quantitative RT‒PCR

RNA was extracted from tissues and cells using TRIzol (Takara, Japan). RNA (1000 ng) was reverse transcribed using a PrimeScript™ RT Reagent Kit (Takara, Japan). Quantitative RT‒PCR was performed with SYBR Green Master Mix (Vazyme, Nanjing, China). 18S rRNA was used as an endogenous control. Relative gene expression levels were calculated with the 2^(− ΔΔCT) method. The primers used were as follows:

KPNA2, forward 5′-CTGCCCGTCTTCACAGATTCA-3′, reverse 5′-GCGGAGAAGTAGCATCATCAGG-3′, ANGPT2, forward 5′-AACTTTCGGAAGAGCATGGAC-3′, reverse 5′-CGAGTCATCGTATTCGAGCGG-3′, VEGFA forward 5′-AGGGCAGAATCATCACGAAGT-3′, reverse 5′-AGGGTCTCGATTGGATGGCA-3′, 18S, forward 5′-TTGACGGAAGGGCACCACCAG-3′, reverse 5′-GCACCACCACCCACGGAATCG-3′.

### Coimmunoprecipitation (Co-IP) assays

Co-IP assays were performed as described previously. The cells were lysed with RIPA lysis buffer (Beyotime Biotechnology, P0013C) supplemented with PMSF (Thermo Scientific) and protease inhibitors (Roche, 04693132001). The lysate was sonicated for 30 s and kept on ice for 1 h, and the supernatant was collected. Mixed 50 µl protein A/G magnetic beads (MedChemExpress, cat# HY-K0202) into the protein-antibody solution and incubated on a shaker at 4 °C for 3 h. The magnetic beads were precipitated with a magnetic stand. The magnetic beads were boiled with SDS loading buffer and subjected to immunoblotting analysis. The magnetic beads were also used for immunoprecipitation-mass spectrometry (IP-MS).

### IP-MS

After sample processing, mass spectrometry data were collected using a Q Exactive Plus mass spectrometer (Thermo Scientific) in series with an EASY-nLC 1200 liquid phase LC/MS system (Thermo Scientific). The mass spectral data were searched with MaxQuant (V1.6.6) software, and the database search algorithm Andromeda was used to search the Human's Proteome Reference Database in UniProt (2020-05-10, containing 75074 protein sequences).

### Statistical analysis

All data are presented as the mean ± SD. Statistical analysis between two groups was carried out by the two-tailed unpaired Student’s t test. All experiments were performed as multiple biological replicates (n = 3–6). Differences for which P < 0.05 were considered to be statistically significant. Statistical analysis was performed using GraphPad Prism 8.0.

## Results

### KPNA2 was upregulated in the HUVEC hypoxia model and murine hindlimb ischemia model

**Fig. 1:**
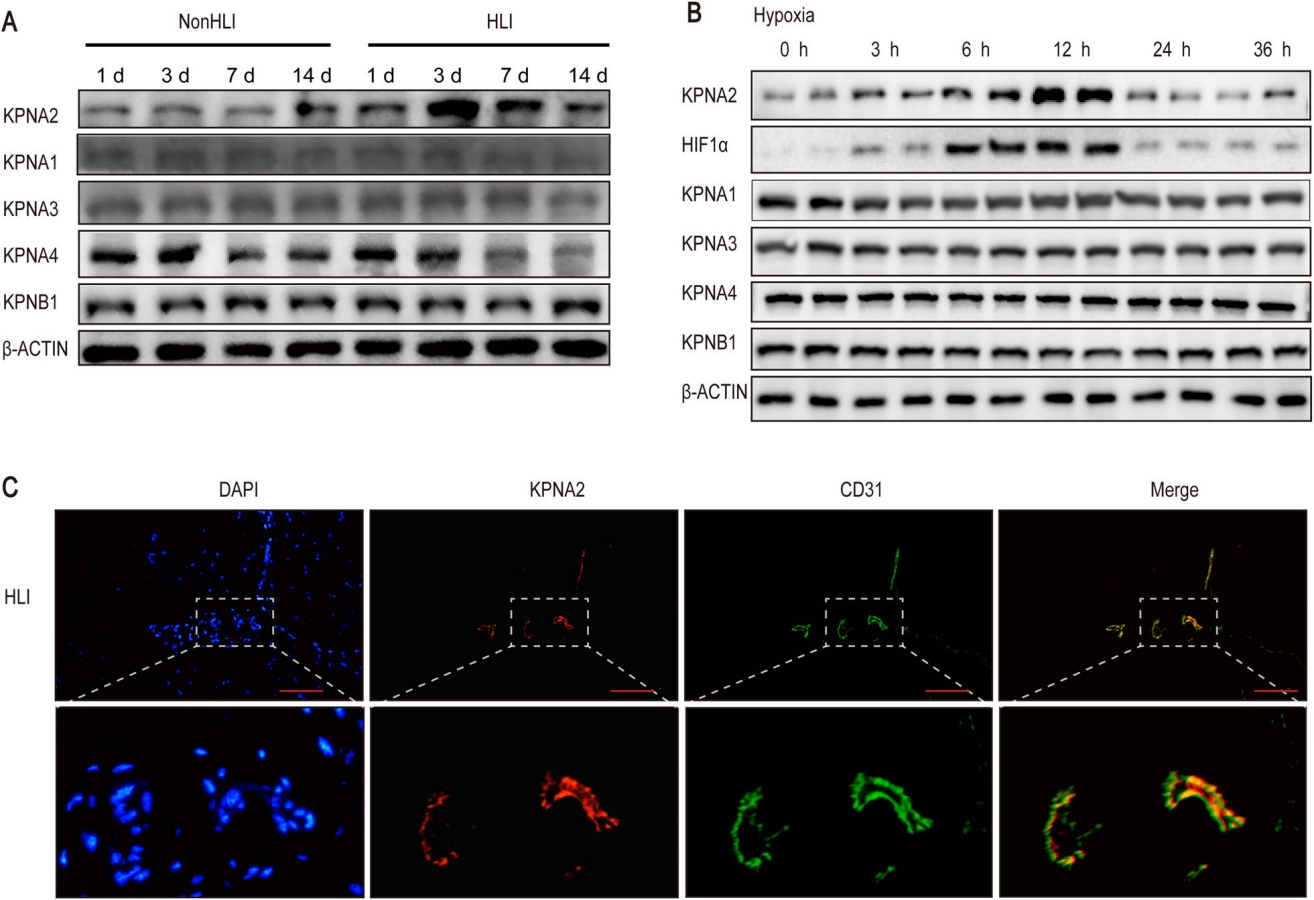
KPNA2 was upregulated in a HUVEC hypoxia model and murine hindlimb ischemia model. **A** The gastrocnemius muscle was taken from the non-ischemic hindlimb and ischemic hindlimb of model mice at 1 days, 3 days, 7 days, and 14 days, and changes in KPNA2, KPNA1, KPNA3, KPNA4, and KPNB1 levels were detected by Western blotting. n = 5. **B** Changes in KPNA2, KPNA1, KPNA3, KPNA4 and KPNB1 levels in HUVEC at 0 h, 6 h, 12 h, 24 h and 36 h of hypoxia were detected by Western blotting. Each experiment was repeated three times. **C** The gastrocnemius muscle was harvested from the ischemic hindlimb of model mice at 14 days for KPNA2 immunofluorescence staining (red), the endothelium was labelled with CD31 (green), while the nuclei (blue) were stained with DAPI. Scale bar: 200 μm. n = 5. *NonHLI* non-hindlimb ischemia, *HLI* hindlimb ischemia

A murine hindlimb ischemia model was constructed by ligating the mouse femoral artery, and the gastrocnemius muscle was taken at 1 days, 3 days, 7 days, and 14 days. The expression level of KPNA2 was higher at 3 days and 7 days and the expression level of KPNA4 was slightly decreased at 14 days, while the expression levels of KPNA1, KPNA3, and KPNB1 did not change (Fig. 1A, Additional file 1: Fig. S1A). In vitro, HUVEC were treated with 1% O_2_, and the KPNA2 protein was detected at 0 h, 3 h, 6 h, 12 h, 24 h and 36 h. The expression of KPNA2 increased of hypoxia treatment at 3 h, 6 h, 12 h, and then decreased at 24 h, 36 h (Fig. 1B, Additional file 1: Fig. S1B). The gastrocnemius muscle was harvested on day 14 of murine hindlimb ischemia. KPNA2 was used for immunofluorescence staining, CD31 was used to mark the endothelium, and DAPI was used to stain the nuclei. The results showed that KPNA2 partly colocalized with the endothelium (Fig. 1C). Therefore, we hypothesized that KPNA2 plays a role in hypoxia-induced angiogenesis.

### *Overexpression of KPNA2 promoted angiogenesis *in vitro* and *in vivo* under hypoxia*

**Fig. 2:**
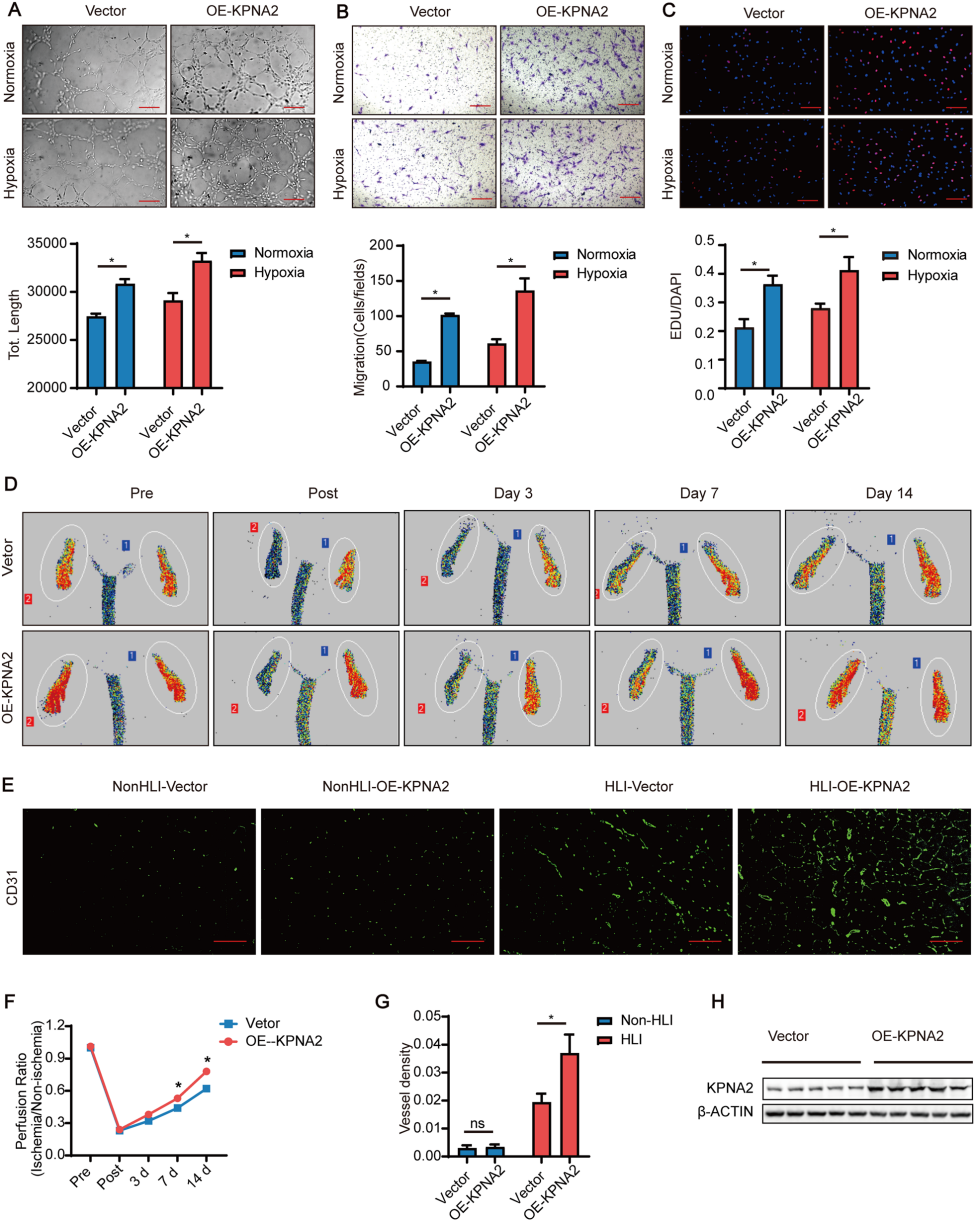
Overexpression of KPNA2 promoted angiogenesis in vitro and in vivo under hypoxia. **A** HUVEC were infected with Ad-KPNA2 to overexpress KPNA2. After 48 h of infection, tube formation assays were carried out under normoxia and under hypoxia, and pictures were taken 6 h later. Scale bar: 200 μm. The total lengths of the tube formation assay were analyzed statistically. Each experiment was repeated three times. **B** HUVEC were infected with Ad-KPNA2 to overexpress KPNA2. After 48 h of infection, Transwell assays were performed under hypoxia, and pictures were taken 6 h later. Scale bar: 200 μm. For each experiment, 3 fields of view were randomly selected for cell counting, and the average value was taken for statistical analysis. Each experiment was repeated three times. **C** HUVEC were infected with Ad-KPNA2 to overexpress KPNA2. After 48 h of infection, EdU staining was carried out under hypoxia, and pictures were taken 6 h later. Scale bar: 200 μm (EDU: red, DAPI: blue). EDU/DAPI were analyzed statistically. Each experiment was repeated three times. **D** Changes in blood perfusion in the ischemic hindlimb of model mice after overexpression of KPNA2 before and after surgery and at 3 days, 7 days, and 14 days. n = 5. **E** CD31 immunofluorescence (green) analysis of the gastrocnemius muscle of the ischemic mouse hindlimb at 14 days after KPNA2 overexpression. Scale bar 100 μm. n = 5. **F** Statistical analysis of blood flow changes in the ischemic hindlimb of model mice after overexpression of KPNA2 before and after surgery and at 3, 7, and 14 days. n = 5. **G** Vessel density statistics of CD31 immunofluorescence of the gastrocnemius muscle of the ischemic mouse hindlimb at 14 days after KPNA2 overexpression. n = 5. **H** KPNA2 expression of the gastrocnemius muscle of the ischemic mouse hindlimb at 14 days after KPNA2 overexpression detected by Western Blot. n = 5. * p < 0.05. *NonHLI* non-hindlimb ischemia, *HLI* hindlimb ischemia

To explore the effect of KPNA2 on the tube formation, proliferation and migration of HUVEC under normoxia and under hypoxia in vitro, Ad-KPNA2 was used to infect HUVEC to overexpress KPNA2, and Ad-Vector was used to infect HUVEC as a control. The effect of KPNA2 on HUVEC tube formation in vitro was detected by tubule formation assay, and the results showed that overexpression of KPNA2 promoted the tube formation of HUVEC under normoxia and under hypoxia (Fig. 2A). The effect of KPNA2 on HUVEC migration was detected by Transwell experiments. Then, the overexpression of KPNA2 promoted the migration of HUVEC under normoxia and under hypoxia (Fig. 2B). The effect of KPNA2 overexpression on HUVEC proliferation was detected by staining with EdU and DAPI. The overexpression of KPNA2 promoted HUVEC proliferation under normoxia and under hypoxia (Fig. 2C).

To explore the effect of KPNA2 on angiogenesis under hypoxia in vivo, a murine hindlimb ischemia model was constructed by ligating the mouse femoral artery, and Ad-KPNA2 was locally injected into the gastrocnemius muscle to overexpress KPNA2 in vivo. Ad-Vector was locally injected into the gastrocnemius muscle in a different mouse as a control. Changes in blood flow in the lower limbs of the mice were detected before and after surgery and at 3 days, 7 days, and 14 days using a laser Doppler flowmeter, and the capillary density of the gastrocnemius muscle of the mice was observed by immunofluorescence staining with CD31. Overexpression of KPNA2 promoted the restoration of ischemic hindlimb blood flow at 7 d and 14 d compared with that in the control group. (Fig. 2D, F) Furthermore, overexpression of KPNA2 significantly increased the capillary density of the ischemic hindlimb compared with the control hindlimb (Fig. 2E, G). Western Blot was used to detect the overexpression efficiency at the protein level, and the results showed that the expression of KPNA2 increased in the overexpression group compared with the control (Fig. 2H, Additional file 1: Fig. S1C).

The above results suggested that overexpression of KPNA2 promoted angiogenesis in vitro and in vivo under hypoxia.

### *Knockdown of KPNA2 inhibited angiogenesis *in vitro* and *in vivo* under hypoxia*

**Fig. 3:**
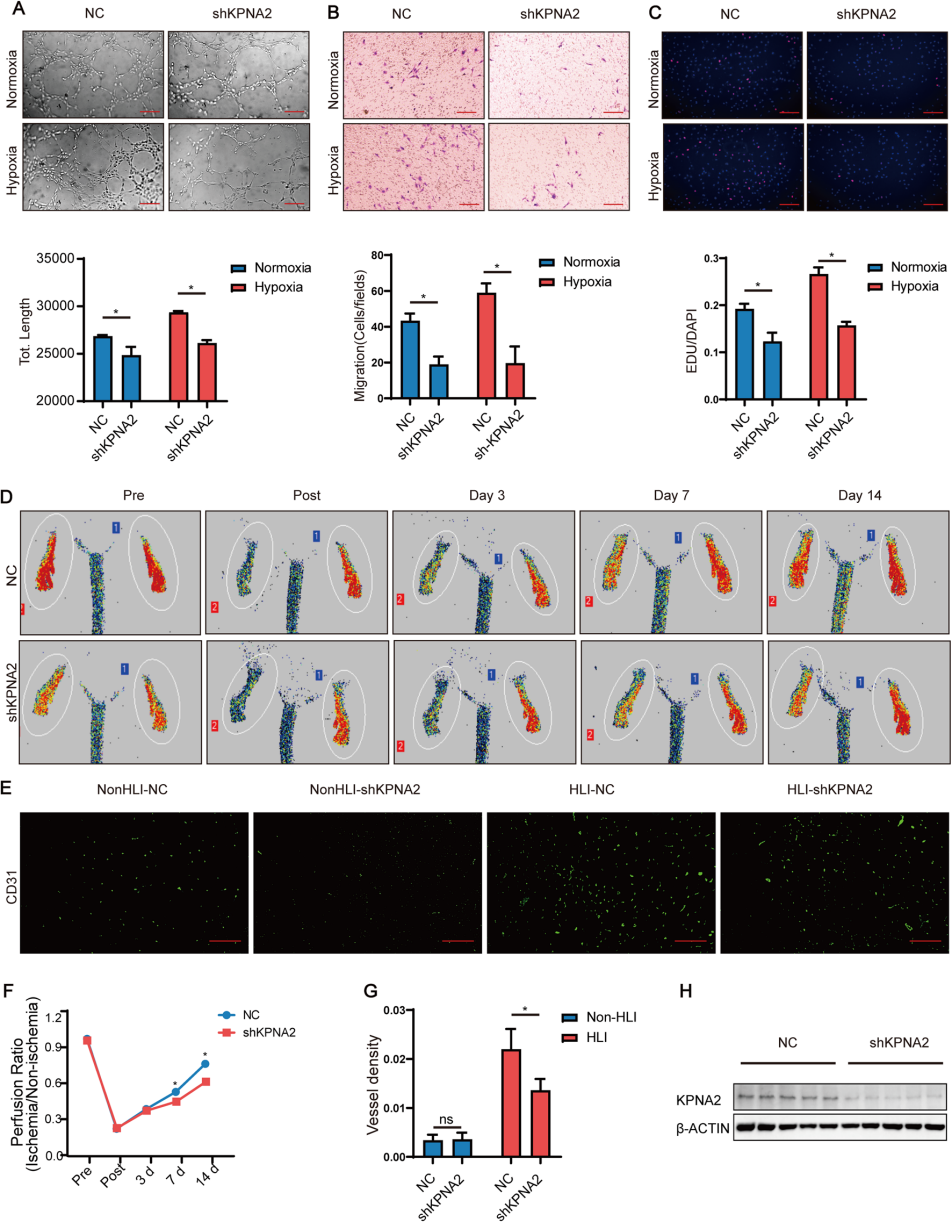
Knockdown of KPNA2 inhibited angiogenesis in vitro and in vivo under hypoxia. **A** HUVEC were infected with Ad-shKPNA2 to knock down KPNA2. After 48 h of infection, tube formation assays were carried out under normoxia and under hypoxia, and pictures were taken 6 h later. Scale bar: 200 μm. The total lengths of the tube formation assay were analyzed statistically. Each experiment was repeated three times. **B** HUVEC were infected with Ad-shKPNA2 to knock down KPNA2. After 48 h of infection, Transwell assays were performed under normoxia and under hypoxia, and pictures were taken 6 h later. Scale bar: 200 μm. For each experiment, 3 fields of view were randomly selected for cell counting, and the average value was taken for statistical analysis. Each experiment was repeated three times. **C** HUVEC were infected with Ad-shKPNA2 to knock down KPNA2. After 48 h of infection, EdU staining was carried out under hypoxia (EdU: red, DAPI: blue), and pictures were taken 6 h later. Scale bar: 200 μm. EDU/DAPI were analyzed statistically. Each experiment was repeated three times. **D** Changes in blood perfusion in the ischemic hindlimb of model mice after KPNA2 knockdown before and after surgery and at 3 days, 7 days and 14 days. n = 5. **E** CD31 immunofluorescence (green) analysis of the gastrocnemius muscle of the ischemic mouse hindlimb at 14 d after KPNA2 knockdown. Scale bar: 100 μm. n = 5. **F** Statistical analysis of blood flow changes in the ischemic hindlimb of model mice after KPNA2 knockdown before and after surgery and at 3 days, 7 days, and 14 days. n = 5. **G** Vessel density statistics of CD31 immunofluorescence of the gastrocnemius muscle of the ischemic mouse hindlimb at 14 days after KPNA2 overexpression. n = 5. **H** KPNA2 expression of the gastrocnemius muscle of the ischemic mouse hindlimb at 14 days after KPNA2 knockdown detected by Western Blot. n = 5. * p < 0.05. *NC* negative control, *NonHLI* non- hindlimb ischemia, *HLI* hindlimb ischemia

Ad-shKPNA2 was used to infect HUVEC to knock down KPNA2, and Ad-NC was used to infect HUVEC as a control. The effect of KPNA2 knockdown on HUVEC tube formation under hypoxia in vitro was detected by tubule formation assay, and the results showed that KPNA2 knockdown inhibited HUVEC tube formation under normoxia and under hypoxia (Fig. 3A). Then, the effect of KPNA2 knockdown on the migration of HUVEC was examined by Transwell experiments. KPNA2 knockdown inhibited the migration of HUVEC under normoxia and under hypoxia (Fig. 3B). Finally, the effect of KPNA2 knockdown on HUVEC proliferation was detected by EdU and DAPI staining. The results showed that KPNA2 knockdown inhibited HUVEC proliferation under normoxia and under hypoxia (Fig. 3C).

Ad-shKPNA2 was locally injected into the gastrocnemius muscle to knock down KPNA2 in vivo, and Ad-NC was locally injected into the gastrocnemius muscle in a different mouse as a control. Changes in blood flow in the hindlimb of the mice were detected at 0 days, 3 days, 7 days, and 14 days using a laser Doppler flowmeter, and the capillary density of the gastrocnemius muscle of the mice was observed by immunofluorescence staining with CD31. The results showed that knockdown of KPNA2 inhibited the restoration of ischemic hindlimb blood flow at 7 days and 14 days compared with that in the control group (Fig. 3D, F). Furthermore, knockdown of KPNA2 significantly decreased the capillary density of the ischemic hindlimb compared with the control hindlimb (Fig. 3E, G). Knockdown of KPNA2 inhibited angiogenesis in vivo.

The above results suggested that knockdown of KPNA2 inhibited angiogenesis in vitro and in vivo under hypoxia. Western Blot was used to detect the knockdown efficiency at the protein level, and the results showed that the expression of KPNA2 in the knockdown group decreased compared with the control. (Fig. 3H, Additional file 1: Fig. S1D).

### The binding of KPNA2 to STAT3 was increased under hypoxia

To explore the mechanism by which KPNA2 promotes angiogenesis under hypoxia, KPNA2 antibody and IgG antibody were used to carry out a Co-IP experiment with HUVEC under hypoxia and normoxia, and the magnetic beads were assessed by IP-MS.

**Fig. 4:**
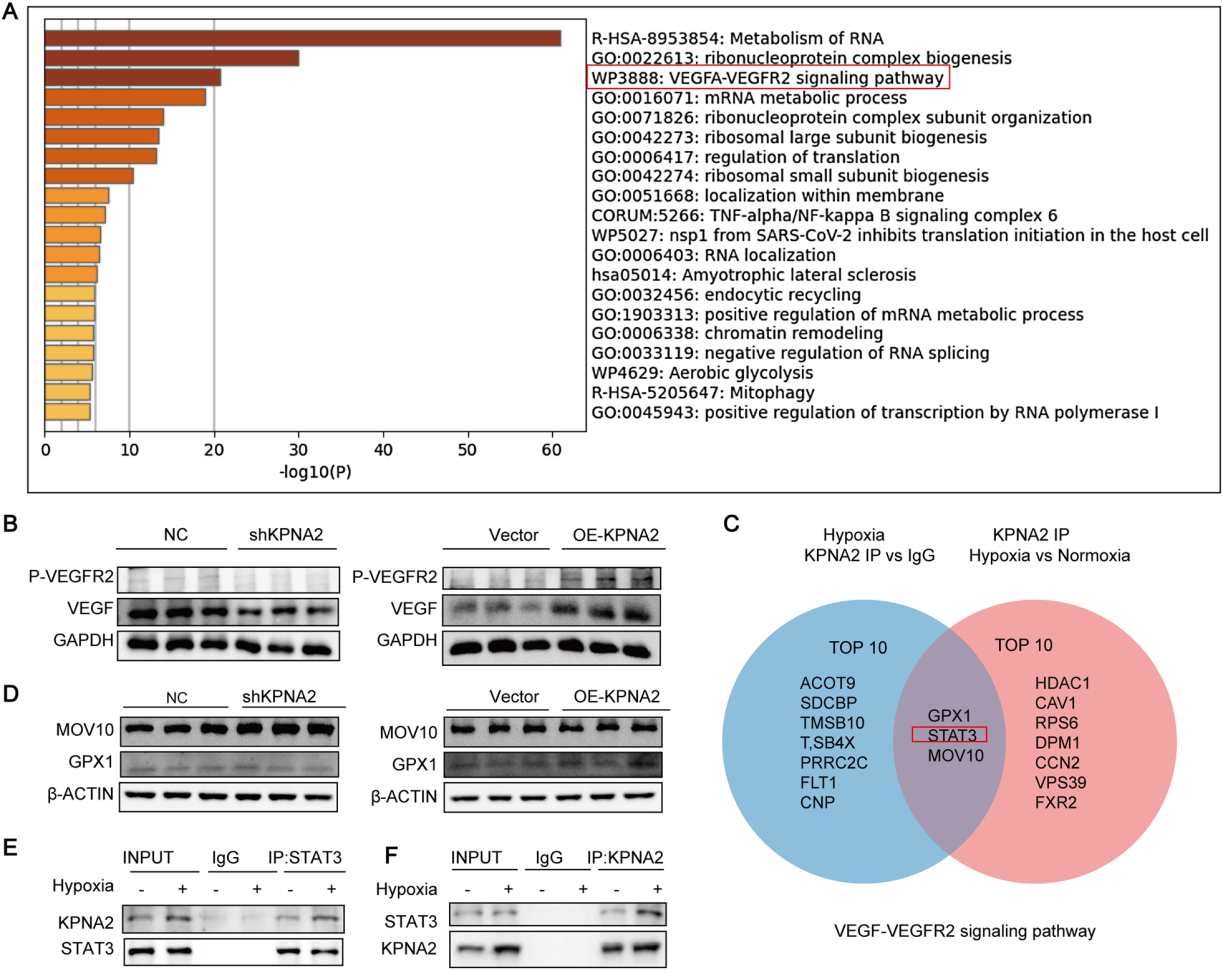
The binding of KPNA2 and STAT3 was increased under hypoxia. **A** IP-MS of KPNA2 showed that the binding of 364 proteins to KPNA2 was increased under hypoxia compared to normoxia. Metascape was used for enrichment analysis of these proteins, and the results showed that the top three enriched pathways were RNA metabolism, ribonucleoprotein complex biogenesis, and the VEGFA-VEGFR2 pathway. **B** After overexpression and knockdown of KPNA2 in HUVEC under hypoxia for 12 h, changes in VEGF and P-VEGFR2 levels were detected by Western blotting, and GAPDH was used as an internal control. **C** We selected the top ten proteins related to the VEGFA-VEGFR2 pathway upregulated under hypoxia (IgG as a control) and the top ten proteins related to the VEGFA-VEGFR2 pathway that were found to be upregulated under hypoxia vs. normoxia identified by IP-MS analysis of KPNA2 in HUVEC and determined the intersecting proteins. We obtained three proteins, namely, GPX1, STAT3 and MOV10. **D** The changes in MOV10 and GPX1 levels were detected by Western blotting after overexpression or knockdown of KPNA2 in HUVEC under hypoxia for 12 h, and β-ACTIN was an internal control. **E** Western blotting analysis of immunoprecipitated STAT3 under normoxia vs hypoxia for 12 h was used to detect KPNA2. **F** IP analysis of KPNA2 under normoxia vs hypoxia for 12 h was carried out, and Western blotting was used to detect STAT3. Each experiment was repeated three times

The results showed that the binding of 364 proteins to KPNA2 was increased under hypoxia compared to normoxia. We used Metascape to perform enrichment analysis of these proteins, and the results showed that the top three enriched pathways were RNA metabolism, ribonucleoprotein complex biogenesis, and the VEGFA-VEGFR2 pathway (Fig. 4A). We believe that the ability of KPNA2 to promote angiogenesis under hypoxia may be related to the VEGFA-VEGFR2 pathway. Ad-KPNA2 and Ad-shKPNA2 were used to infect HUVEC that were treated with 1% O_2_ hypoxia, and changes in VEGF and p-VEGFR2 levels were detected by Western blotting. The expression of VEGF and the phosphorylation of VEGFR2 increased when KPNA2 was overexpressed, while the expression of VEGF and the phosphorylation of VEGFR2 decreased when KPNA2 was knocked down (Fig. 4B, Additional file 1: Fig. S1E, F). To identify the proteins that play an important role in this process, the top ten proteins related to the VEGFA-VEGFR2 pathway identified by Co-IP analysis of KPNA2 in hypoxic HUVEC and the top ten proteins related to the VEGFA-VEGFR2 pathway that were found to be upregulated under hypoxia vs normoxia by Co-IP analysis of KPNA2 in HUVEC were selected, and the intersecting proteins were identified. Three proteins were obtained, namely, glutathione peroxidase 1 (GPX1), STAT3 and Mov10 RISC complex RNA helicase (MOV10) (Fig. 4C). The changes in GPX1 and MOV10 expression after KPNA2 overexpression and knockdown were detected by Western blotting, which showed no changes in GPX1 or MOV10 after KPNA2 overexpression or knockdown (Fig. 4D, Additional file 1: Fig. S1E, F). Changes in the binding of KPNA2 to STAT3 in HUVEC under normoxia and hypoxia were validated by Western blotting. Consistent with the results of IP-MS, the binding of KPNA2 to STAT3 was increased under both 12 h and 24 h hypoxia (Fig. 4E, F, Additional file 1: Fig. S1G, Additional file 2: Fig. S2A).

### KPNA2 promotes STAT3 phosphorylation and the nuclear accumulation of phosphorylated STAT3

**Fig. 5:**
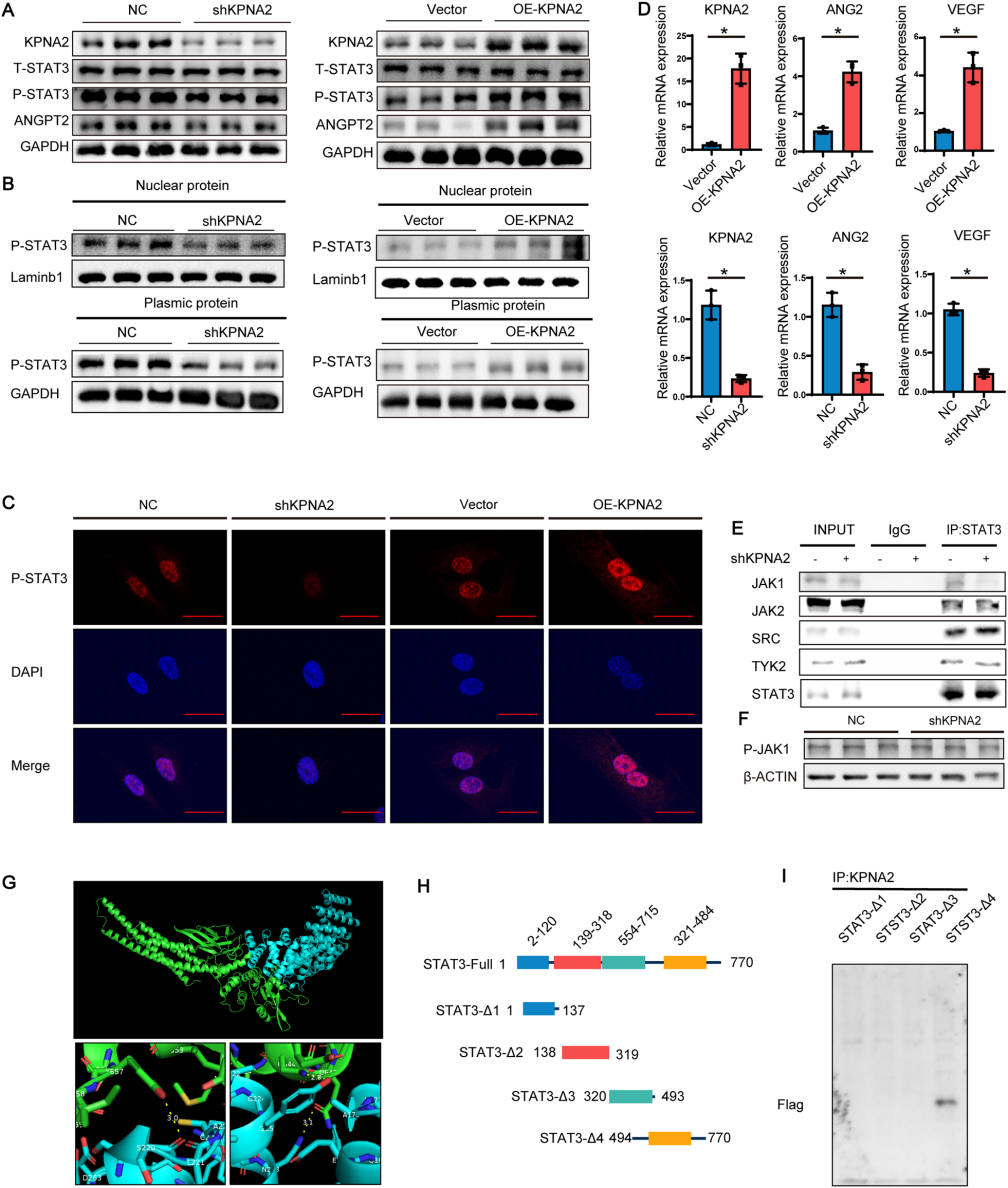
KPNA2 promotes STAT3 phosphorylation and the nuclear accumulation of phosphorylated STAT3. **A** Western blot analysis of the changes in KPNA2, STAT3, P-STAT3 and ANPTG2 levels in HUVEC under hypoxia for 12 h after overexpression and knockdown of KPNA2. GAPDH was used as an internal reference. **B** KPNA2 was overexpressed and knocked down in HUVEC under hypoxia for 12 h, and P-STAT3 was detected by Western blotting after separation of the nuclear and cytoplasmic proteins. The nuclear reference was Laminb1, and the cytoplasmic reference was GAPDH. **C** Immunofluorescence detection of P-STAT3 (red) and DAPI staining of the nuclei (blue) under hypoxia for 12 h after KPNA2 was overexpressed or knocked down in adenovirus-infected HUVEC. Scale bar: 25 μm **D** Quantitative RT-PCR detection of VEGF and ANGPT2 mRNA expression under hypoxia in HUVEC in which KPNA2 was overexpressed or knocked down. **E** After adenovirus infection of HUVEC to knock down KPNA2 under hypoxia for 12 h, STAT3 was assessed by Co-IP, and Western blotting was used to detect JAK1, JAK2, SRC, TYK2, and STAT3. **F** KPNA2 was knocked down in HUVEC under hypoxia for 12 h, and P-JAK1 was detected by Western blotting. **G** Molecular simulations with ZDOCK showed possible interactions between S220 (KPNA2)-Y657 (STAT3), N228 (KPNA2)-Q643 (STAT3), and Y225 (KPNA2)-Q644 (STAT3). **H** Constructs from truncated plasmids corresponding to 4 domains of STAT3 with added Flag tags were prepared; the constructs corresponded to amino acids 1–137, 138–319, 320–493, and 494–770. **I** Four truncated STAT3 plasmids were transfected into 293 T cells, KPNA2 was used for Co-IP, and Flag was detected by Western blotting. Each experiment was repeated three times. *NC* negative control

Changes in STAT3 content and phosphorylation in HUVEC under hypoxia after KPNA2 overexpression and knockdown were detected by Western blotting. The results showed no change in the amount of total STAT3 after overexpression or knockdown of KPNA2. Overexpression of KPNA2 promoted the phosphorylation of STAT3 under hypoxia, and knockdown of KPNA2 inhibited the phosphorylation of STAT3 (Fig. 5A, Additional file 1: Fig. S1E, F).

To explore the effect of KPNA2 on the nucleocytoplasmic distribution of P-STAT3 in HUVEC under hypoxia, the nucleocytoplasmic proteins of HUVEC in which KPNA2 was overexpressed or knocked down under hypoxia were separated, and P-STAT3 was detected in the nucleus and cytoplasm by Western blotting. The levels of P-STAT3 in both the nucleus and cytoplasm increased after overexpression of KPNA2 and decreased after knockdown of KPNA2 (Fig. 5B, Additional file 1: Fig. S1H).

Immunofluorescence staining of P-STAT3 was performed, and the nuclei were stained with DAPI, which showed that P-STAT3 was increased in both the nucleus and cytoplasm (Fig. 5C). STAT3, a transcription factor, can regulate the expression of VEGFA and ANGPT2, and VEGFA and ANGPT2 play an important role in angiogenesis. The expression of ANGPT2 after overexpression or knockdown of KPNA2 under hypoxia was detected by Western blotting, and the results showed increased expression of ANGPT2 after overexpression of KPNA2 and decreased expression of ANGPT2 after knockdown of KPNA2 (Fig. 5A). The quantitative RT‒PCR results showed that the relative mRNA expression of VEGF and ANGPT2 increased after KPNA2 overexpression, while the relative mRNA expression of VEGF and ANGPT2 decreased after KPNA2 knockdown (Fig. 5D).

To explore how KPNA2 affects the phosphorylation of STAT3, Ad-shKPNA2 was used to infect HUVEC to knock down KPNA2 under hypoxia. Co-IP experiment was performed with anti-STAT3 antibody, and the binding of STAT3 to the phosphorylated kinases JAK1, JAK2, TYK2, and SRC was detected by Western blotting. The results showed that KPNA2 knockdown inhibited the binding of STAT3 to JAK1 in HUVEC under hypoxia (Fig. 5E, Additional file 1: Fig. S1I). P-JAK1 levels did not change after KPNA2 knockdown (Fig. 5F). The above results suggested that KPNA2 promoted the phosphorylation and nuclear import of STAT3 by promoting the binding of STAT3 to JAK1.

To further investigate which domain of STAT3 binds KPNA2, structural prediction was performed with ZDOCK. The results showed that KPNA2 bound the SH2 domain of STAT3 and that these proteins likely bind at the following residue pairs: S220 (KPNA2)-Y657 (STAT3), N228 (KPNA2)-Q643 (STAT3), and Y225 (KPNA2)-Q644 (STAT3) (Fig. 5G). Truncated plasmids corresponding to the four domains of STAT3 (amino acids 1–137, 138–319, 320–493, and 494–770) were constructed. (Fig. 5H) The plasmids were transfected into 293 T cells, and anti-KPNA2 antibody was used for a Co-IP experiment, while anti-flag antibody was used for Western blotting. The results showed that KPNA2 bound the SH2 domain of STAT3 (Fig. 5I).

### A STAT3 inhibitor reversed the ability of KPNA2 to promote angiogenesis

**Fig. 6:**
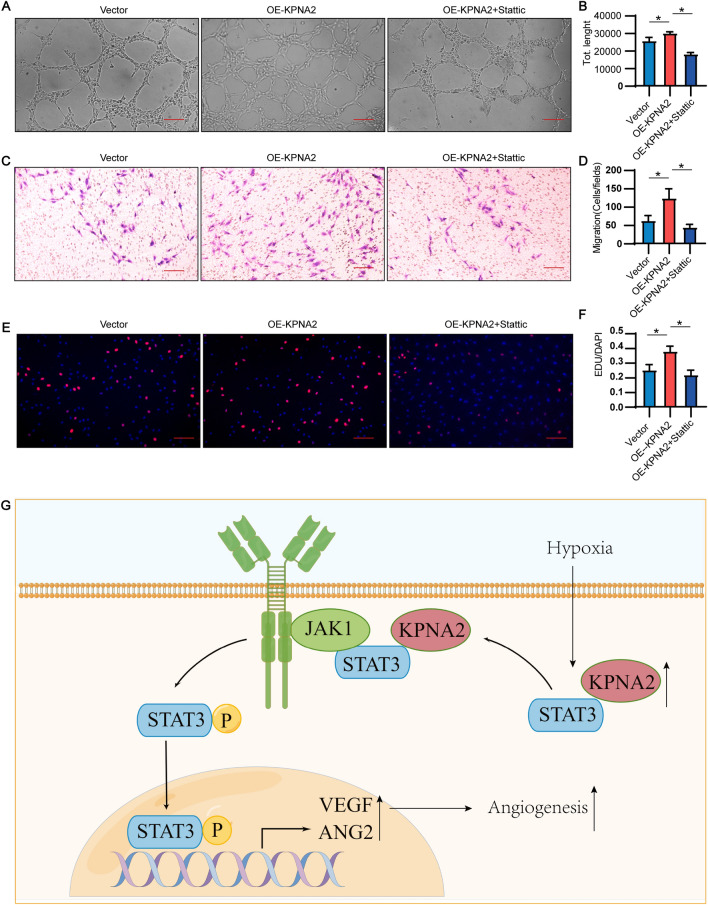
A STAT3 inhibitor reversed the ability of KPNA2 to promote angiogenesis in vitro. **A**Adenovirus-infected HUVEC overexpressed KPNA2 and were treated with a STAT3 inhibitor, and tube formation assays were carried out under hypoxia. Images were taken after 6 h. Scale bar: 200 μm. **B** Total tube length from tube formation assays. **C** Adenovirus-infected HUVEC overexpressed KPNA2 and were treated with a STAT3 inhibitor before Transwell assays performed under hypoxia. Images were taken after 6 h. Scale bar: 200 μm. **D** Statistical analysis of the results of Transwell assays (cells/fields). **E** Adenovirus-infected HUVEC overexpressing KPNA2 were treated with a STAT3 inhibitor and subjected to EdU/DAPI staining under hypoxia (red: EdU, blue: DAPI). Scale bar: 200 μm. **F** Statistical analysis of the EdU/DAPI staining results. **G** The binding of STAT3 and KPNA2 promotes the binding of STAT3 and JAK1 and phosphorylation of STAT3. P-STAT3 enters the nucleus, where it promotes the expression of VEGF and ANGPT2, which promotes angiogenesis. Each experiment was repeated three times. * p < 0.05

To verify that the ability of KPNA2 to promote angiogenesis occurs through STAT3, an inhibitor of STAT3, Stattic, was used for an in vitro recovery experiment. The results showed that the increases in the tube formation, proliferation and migration of HUVEC induced by KPNA2 overexpression under hypoxia could be reversed by the STAT3 inhibitor (Fig. 6A–F).

The above results suggested that KPNA2 promotes the phosphorylation and nuclear import of STAT3 by promoting the binding of STAT3 to JAK1, thereby promoting the expression of VEGFA and ANGPT2 (Fig. 6G).

## Discussion

In this study, we demonstrate that KPNA2 promotes angiogenesis in vivo and in vitro under hypoxic conditions by promoting the phosphorylation of STAT3 and promoting expression of the angiogenic growth factors VEGF and ANGPT2.

KPNA2 has generally been described in previous studies as a transporter that mediates the nuclear transport of proteins including IRF3, P65, E2F1, and c-Myc [28–31]. However, an increasing number of studies have suggested that KPNA2 is a multifunctional protein with a variety of functions in addition to its role in nuclear transport [16]. Recent studies have found that KPNA2 is localized on the surface of various tumour cell types [32]. KPNA2 promotes the phosphorylation of AKT and GSK-3β in tumour cells without altering the expression of total AKT or GSK3β [33]. Our study showed that KPNA2 can regulate the phosphorylation of STAT3 without changing the total amount of STAT3, thereby promoting the nuclear entry of P-STAT3 and upregulating the downstream target genes STAT3 VEGF and ANGPT2. These studies suggest that KPNA2 is involved in regulating protein phosphorylation, which provides new ideas for future research on KPNA2.

The relationship between KPNA2 and STAT3 is controversial. Some studies have shown that KPNA2 and STAT3 colocalize in the nucleoplasm of tumour cells [34], and IP results in fibroblast-like synoviocytes suggest that KPNA2 binds STAT3 [35]. Immunofluorescence analysis showed reduced STAT3 nuclear entry after KPNA2 knockdown in pancreatic ductal adenocarcinoma cells [36]. However, some studies have also shown that STAT3 enters the nucleus by binding KPNA3 rather than KPNA2 [37]. Our IP-MS and IP-Western blot results indicated that the binding of KPNA2 to STAT3 in endothelial cells was increased under hypoxia, but we did not examine the specific mechanism related to this change. Previous studies have shown that posttranslational modification of KPNA2, such as its phosphorylation and palmitoylation, can modulate its activity and differences in substrate binding [23, 38, 39]. However, whether hypoxia modulates KPNA2 activity and substrate binding by altering the posttranslational modification of KPNA2 requires further study.

STAT3 plays an important role in angiogenesis [40, 41], and its main mechanism involves the upregulated expression of VEGF and ANGPT2 [42–49]. VEGF is mainly involved in endothelial tube formation through VEGFR2 [50], and unlike VEGF, ANGPT2 functions on the stalk and tip cells among endothelial cells [51, 52]. Inhibition of ANGPT2 and VEGF has a cumulative effect on tumour growth and angiogenesis [50, 53]. Our study showed that KPNA2 can simultaneously upregulate the expression of VEGF and ANGPT2 by promoting STAT3 phosphorylation.

STAT3 includes an N-terminal coiled-coil domain, a DNA-binding domain, a linker, an SH2 domain, and a C-terminal transactivation domain. The SH2 domain, which is located between amino acid residues 600 and 700, is essential for the recruitment of STATs to phosphorylated receptors [54]. Our IP results indicated that KPNA2 can bind the SH2 domain of STAT3 and promote the phosphorylation of STAT3. Previous studies have shown that cell density can affect the phosphorylation level of STAT3 [55, 56]. Therefore, to examine the effect of KPNA2 itself on STAT3 phosphorylation, we collected cellular proteins at different cell densities. The Western blotting results showed that the STAT3 phosphorylation level was higher in the KPNA2 overexpression group than in the control group at all cell densities (Additional file 3: Fig. S3A). Moreover, as the degree of KPNA2 overexpression increased, the level of STAT3 phosphorylation also increased (Additional file 3: Fig. S3B). These results supported that KPNA2 itself promoted phosphorylation of STAT3 rather than being cell density dependent.

The binding of STAT3 to JAK1 was reduced after KPNA2 knockdown, while the binding of STAT3 to other phosphorylated kinases, such as JAK2, TYK2, and SRC, was unchanged. These results suggest that the binding of KPNA2 to the SH2 domain of STAT3 promotes the binding of STAT3 to JAK1, thereby promoting the phosphorylation and nuclear import of STAT3.

Briefly, our study shows that under hypoxic conditions, the expression of KPNA2 was upregulated in a hindlimb ischemia model and endothelial hypoxia and that the binding of KPNA2 and STAT3 was increased under hypoxic conditions, which promoted angiogenesis under hypoxic conditions. Mechanistically, we found that KPNA2 binds STAT3 and promotes the binding of JAK1 to STAT3 and the phosphorylation of STAT3 to upregulate VEGF and ANGPT2. However, our research still has some limitations. We used a murine hindlimb ischemia model for our experiments, and the results have not been validated in other models. Furthermore, we used intramuscular adenovirus injection for gene overexpression and knockdown in the animal experiments. The endothelial specificity of intramuscular adenovirus injection is insufficient, so it is impossible to determine whether the effect of KPNA2 was endothelium specific.

In general, our study has clarified the role of KPNA2 in endothelial angiogenesis and provided some insights into the mechanism of angiogenesis under hypoxic conditions and the treatment of angiogenesis-related diseases.

## Supplementary Information

**Additional file 1: Fig. S1.** Relative quantitative statistics of protein expression in Western Blot. (A) Relative quantitative statistics of protein expression of KPNA2, KPNA1, KPNA3, KPNA4, and KPNB1 levels in the gastrocnemius muscle of the ischemic hindlimb of model mice at 1 d, 3 d, 7 d, and 14 d. (B) Relative quantitative statistics of protein expression of KPNA2, KPNA1, KPNA3, KPNA4 and KPNB1 in HUVEC at 0 h, 6 h, 12 h, 24 h and 36 h of hypoxia. (C) Relative quantitative statistics of protein expression of KPNA2 in the gastrocnemius muscle of the ischemic hindlimb of model mice at 14 d after overexpression of KPNA2 (D) Relative quantitative statistics of protein expression of KPNA2 in the gastrocnemius muscle of the ischemic hindlimb of model mice at 14 d after knockdown of KPNA2 (E) Relative quantitative statistics of protein expression of KPNA2, P-VEGF, VEGF, MOV10, GPX1, T-STAT3, ANGPT2, P-JAK1 in HUVEC after knockdown of KPNA2 under hypoxia for 12 h. (F) Relative quantitative statistics of protein expression of KPNA2, P-VEGF, VEGF, MOV10, GPX1, T-STAT3, ANGPT2, P-JAK1 in HUVEC after overexpression of KPNA2 under hypoxia for 12h. (G) Relative quantitative statistics of protein expression of KPNA2 in IP analysis of STAT3 under hypoxia vs normoxia for 12 h. Relative quantitative statistics of protein expression of STAT3 in IP analysis of KPNA2 under hypoxia vs normoxia for 12 h. (H) Relative quantitative statistics of P-STAT3 of the nuclear and cytoplasmic proteins after overexpressed or knocked down KPNA2 in HUVEC under hypoxia for 12 h. (I) Relative quantitative statistics of protein expression of JAK1, JAK2, SRC, TYK2 in IP analysis of STAT3 after knockdown of KPNA2. Each experiment was repeated three times. * p<0.05. NonHLI: non-hindlimb ischemia, HLI: hindlimb ischemia, NC: negative control.

**Additional file 2: Fig. S2.** Binding of KPNA2 to STAT3 was increased under hypoxia for 24 h relative to normoxia. (A)Western blotting analysis was used to detect KPNA2 of IP STAT3 hypoxia for 24 h vs under normoxia. Relative quantitative statistical analysis was performed on protein expression levels. Each experiment was repeated three times. Each experiment was repeated three times. * p<0.05.

**Additional file 3: Fig. S3.** The phosphorylation level of STAT3 is proportional to the expression of KPNA2, and this change is not dependent on the degree of cell confluence. (A) HUVEC were infected with different amounts of Ad-KPNA2 to examine the effect of overexpression of KPNA2 to different degrees, and the protein expression levels of KPNA2, P-STAT3, and STAT3 were detected by Western Blot. Relative quantitative statistical analysis was performed on protein expression levels. (B) Overexpression of KPNA2 at different degrees of cell confluence increased the level of P-STAT3. STAT3 expression levels did not change. Relative quantitative statistical analysis was performed on protein expression levels. Each experiment was repeated three times. * p<0.05.

## Data Availability

Data are available from the corresponding author upon reasonable request.
