# Supplementary figures and images for: KPNA2 promotes angiogenesis by regulating STAT3 phosphorylation
Source: J Transl Med. 2022 Dec 28;20:627. doi: 10.1186/s12967-022-03841-6 (PMC9798605; doi:10.1186/s12967-022-03841-6)

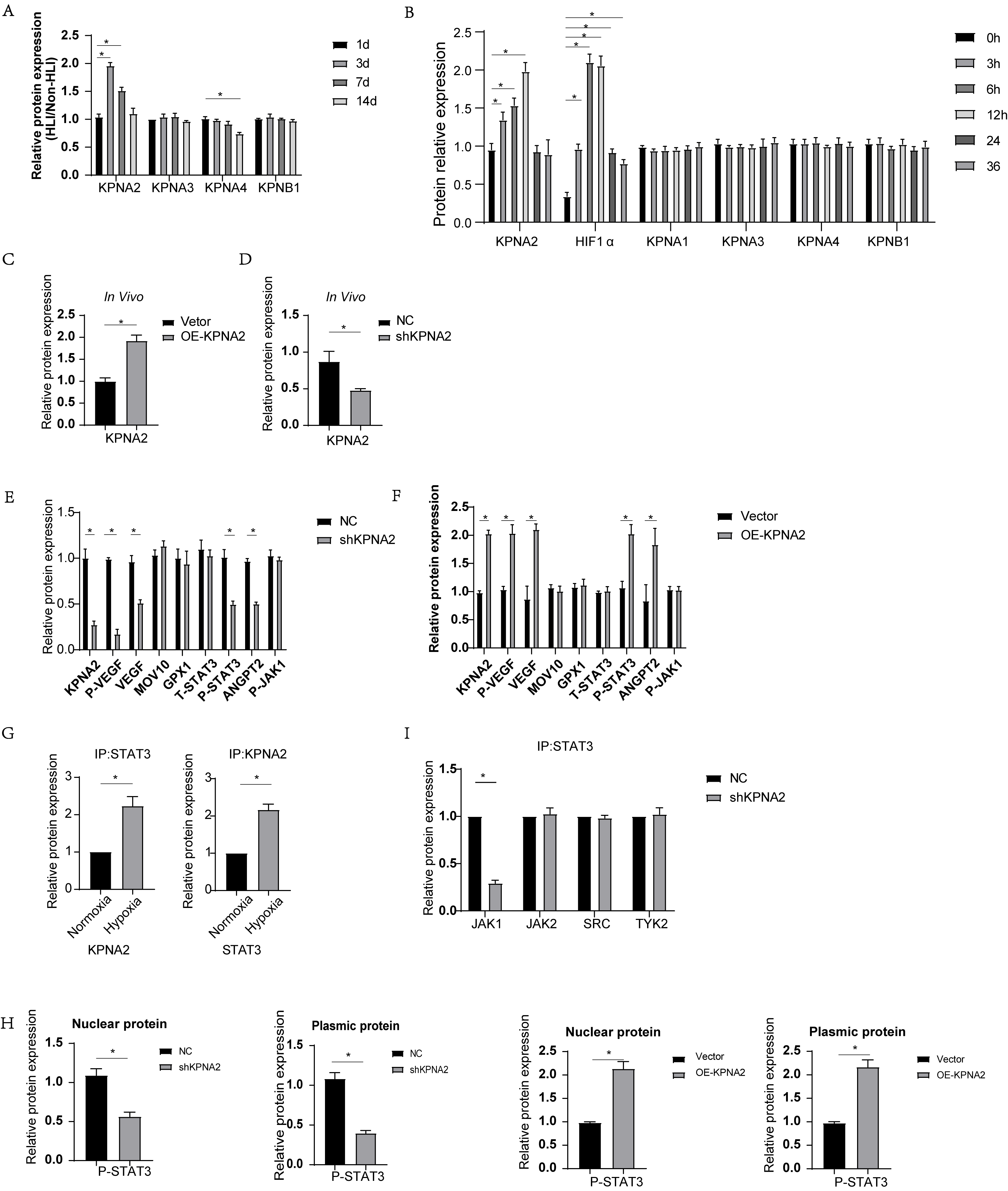

Supplement: Supplementary file 1 — Additional file 1: Fig. S1. Relative quantitative statistics of protein expression in Western Blot. (A) Relative quantitative statistics of protein expression of KPNA2, KPNA1, KPNA3, KPNA4, and KPNB1 levels in the gastrocnemius muscle of the ischemic hindlimb of model mice at 1 d, 3 d, 7 d, and 14 d. (B) Relative quantitative statistics of protein expression of KPNA2, KPNA1, KPNA3, KPNA4 and KPNB1 in HUVEC at 0 h, 6 h, 12 h, 24 h and 36 h of hypoxia. (C) Relative quantitative statistics of protein expression of KPNA2 in the gastrocnemius muscle of the ischemic hindlimb of model mice at 14 d after overexpression of KPNA2 (D) Relative quantitative statistics of protein expression of KPNA2 in the gastrocnemius muscle of the ischemic hindlimb of model mice at 14 d after knockdown of KPNA2 (E) Relative quantitative statistics of protein expression of KPNA2, P-VEGF, VEGF, MOV10, GPX1, T-STAT3, ANGPT2, P-JAK1 in HUVEC after knockdown of KPNA2 under hypoxia for 12 h. (F) Relative quantitative statistics of protein expression of KPNA2, P-VEGF, VEGF, MOV10, GPX1, T-STAT3, ANGPT2, P-JAK1 in HUVEC after overexpression of KPNA2 under hypoxia for 12h. (G) Relative quantitative statistics of protein expression of KPNA2 in IP analysis of STAT3 under hypoxia vs normoxia for 12 h. Relative quantitative statistics of protein expression of STAT3 in IP analysis of KPNA2 under hypoxia vs normoxia for 12 h. (H) Relative quantitative statistics of P-STAT3 of the nuclear and cytoplasmic proteins after overexpressed or knocked down KPNA2 in HUVEC under hypoxia for 12 h. (I) Relative quantitative statistics of protein expression of JAK1, JAK2, SRC, TYK2 in IP analysis of STAT3 after knockdown of KPNA2. Each experiment was repeated three times. * p<0.05. NonHLI: non-hindlimb ischemia, HLI: hindlimb ischemia, NC: negative control. [file 12967_2022_3841_MOESM1_ESM.tif]

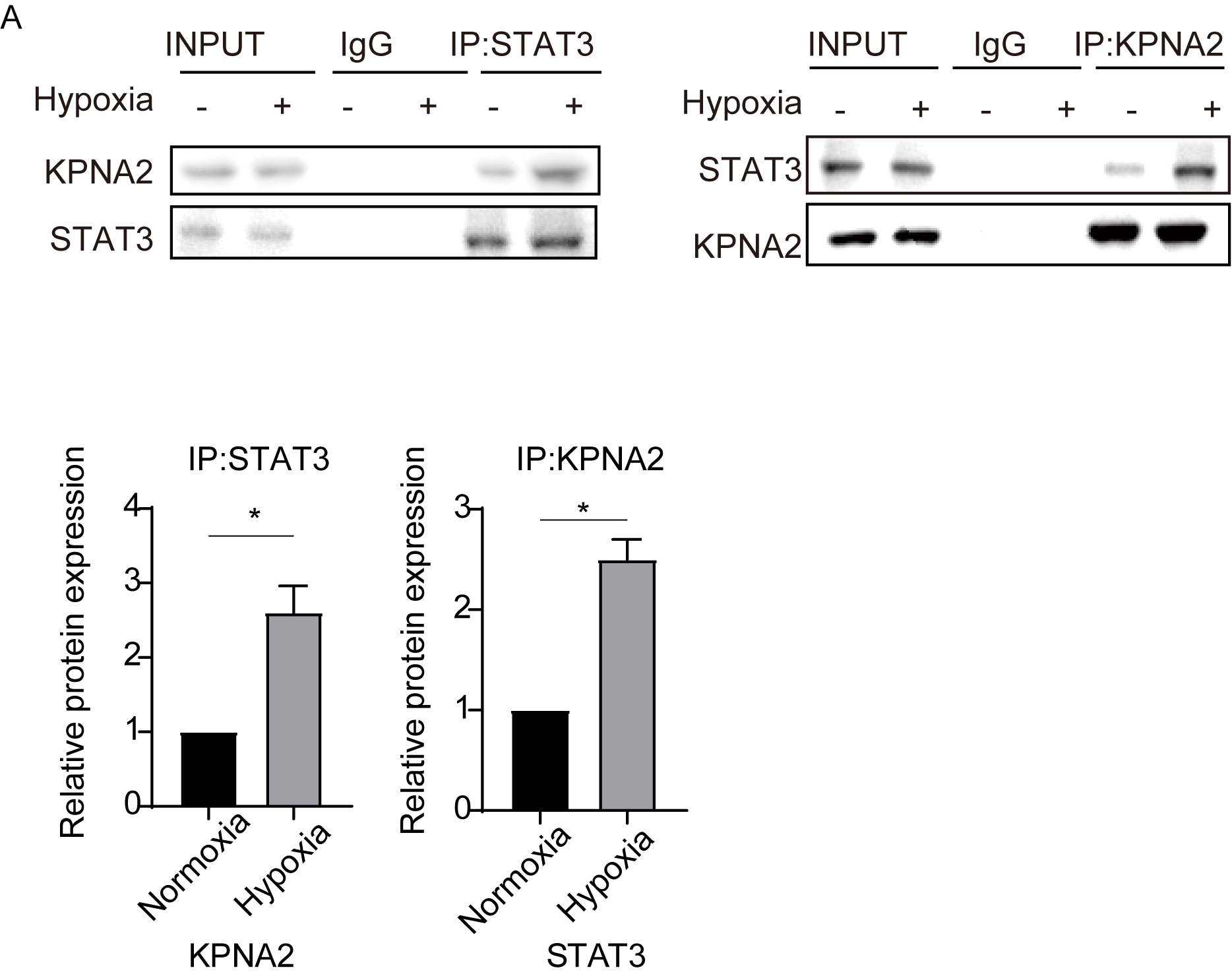

Supplement: Supplementary file 2 — Additional file 2: Fig. S2. Binding of KPNA2 to STAT3 was increased under hypoxia for 24 h relative to normoxia. (A)Western blotting analysis was used to detect KPNA2 of IP STAT3 hypoxia for 24 h vs under normoxia. Relative quantitative statistical analysis was performed on protein expression levels. Each experiment was repeated three times. Each experiment was repeated three times. * p<0.05. [file 12967_2022_3841_MOESM2_ESM.tif]

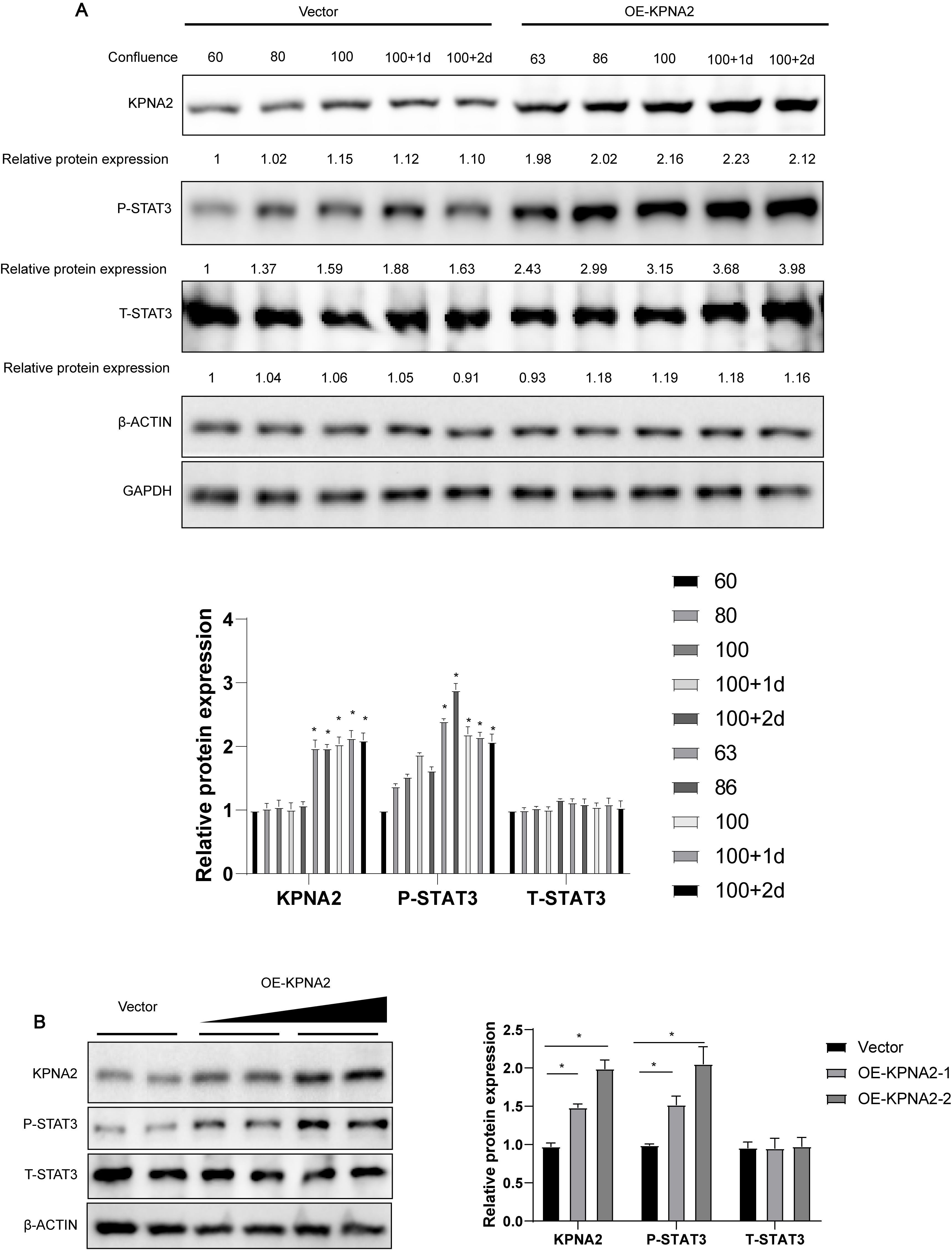

Supplement: Supplementary file 3 — Additional file 3: Fig. S3. The phosphorylation level of STAT3 is proportional to the expression of KPNA2, and this change is not dependent on the degree of cell confluence. (A) HUVEC were infected with different amounts of Ad-KPNA2 to examine the effect of overexpression of KPNA2 to different degrees, and the protein expression levels of KPNA2, P-STAT3, and STAT3 were detected by Western Blot. Relative quantitative statistical analysis was performed on protein expression levels. (B) Overexpression of KPNA2 at different degrees of cell confluence increased the level of P-STAT3. STAT3 expression levels did not change. Relative quantitative statistical analysis was performed on protein expression levels. Each experiment was repeated three times. * p<0.05. [file 12967_2022_3841_MOESM3_ESM.tif]
